# Socio-economic inequality in anaemia among men in India: a study based on cross-sectional data

**DOI:** 10.1186/s12889-021-11393-5

**Published:** 2021-07-07

**Authors:** Pradeep Kumar, Himani Sharma, Debashree Sinha

**Affiliations:** 1 Department of Mathematical Demography & Statistics, International Institute for Population Sciences, Mumbai, Maharashtra 400088 India; 2 Department of Development Studies, International Institute for Population Sciences, Mumbai, Maharashtra 400088 India

**Keywords:** Anaemia, Men, Socio-economic inequality, Concentration index, India

## Abstract

**Background:**

Undernutrition is a serious matter of public health concern in India. Existing studies, policies and programs focus on women and children thereby ignoring men in policymaking. This study examines the socio-economic inequality in anaemia levels among men in India and tries to decompose the factors behind it.

**Methods:**

The fourth round of National Family Health Survey is used to fulfill the study objectives. The outcome variable of the study is men having anaemia or not. The study uses bivariate and multivariate techniques to identify the factors associated with the outcome variable. Further, concentration index and concentration curve are calculated to measure the socio-economic inequality in anaemia among men in India.

**Results:**

The results indicate that majority of the socio-economic related inequality is explained by wealth quintile followed by geographical regions of India, body mass index and educational attainment. The results also emphasize that older men belong to the high-risk groups. Moreover, the likelihood of anaemia is 40% more likely among men who belonged to East region and 25%, 13% and 7 % less likely among those who belonged to Northeast, West and South region compared to those who belonged in the North region of the country.

**Conclusion:**

Existing policies on anaemia should include men to achieve an anaemia free India. Individual education and awareness should be encouraged to improve nutritional status.

## Background

Despite the necessary provisions taken to improve the indicators of nutrition and related health matters over the past few decades, the problem of undernutrition remains as a serious matter of public health concern in the underdeveloped and developing nations. The problem of malnutrition and undernutrition is multifaceted, comprising of stunting, wasting, overweight and anaemia, gripping its force on the global community. According to the Global Nutrition Report 2017, 125 countries face the burden of anaemia wherein, six countries with anaemia only; 38 with anaemia and stunting; 52 with anaemia and overweight; 29 with anaemia, stunting and overweight [1]. The burden of anaemia affects 27% of the world’s population in which the developing countries alone account for more than 89% of the burden [2]. A leading data and analytics company GlobalData, in its epidemiological analysis, revealed that India has the highest prevalence of anaemia at 39.86% among the 16 major pharmaceutical markets [3]. As per the estimates of the World Health Organization (WHO), anaemia is a significant public health problem among 12.7% of the males globally [4]. In addition to this, according to the National Family Health Survey-4, anaemia prevalence was 23% in 2015–16 among men in India, a bare change from 24% in 2005–06 [5]. The situation of anaemia stands alarming not just in India but also in other neighboring countries such as Myanmar, Cambodia, Bangladesh and Nepal as well [6].

In these middle income countries of South Asia, anaemia hampers the economic productivity to a certain extent [7]. However, studies have found inconsistent association between economic growth and reduction of malnutrition [8]. According to Alderman & Linnemayr, the rates of anemia do get influenced by economic growth but the increment is ‘anaemic’ in itself. Despite vigorous economic developments, the rate of decline of anaemia is modest. Although the rates of anemia in the population do decrease as the income increases, the decline is sluggish [7].

Anaemia is a grave public health concern affecting all the segments of society. It is generally defined as a condition in which the hemoglobin concentration is less than a defined level, which subsequently results in the decreased oxygen-carrying capacity of blood [9]. Anaemia in women is a serious cause of concern as blood loss, maternity problems and many more. Similarly, it hampers cognitive development in children. However, in the case of men, anaemia is not recognized as a disease or a significant problem due to concealed and ambiguous symptoms [10].

**Fig. 1:**
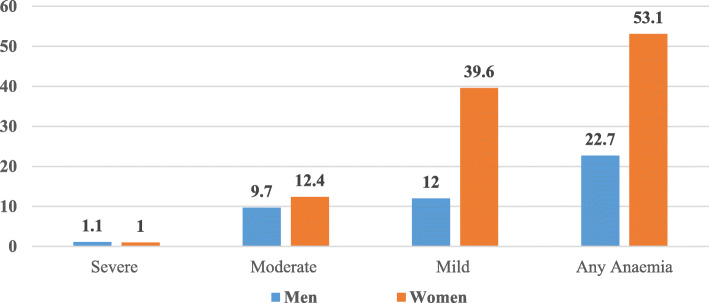
Prevalence of anaemia in men and women age 15–49, India, NFHS, 2015–16

An approximate of a quarter of men aged 15–54 reported having anaemia, both in India in general and in the EAG states, indicating it is as considerable a problem as it is among women and children. Figure 1 depicts the prevalence of anaemia in men and women in the year 2015–16. It is evident that women are more anaemic than men, though the prevalence of anemia among men has also been high enough to be treated as a public health issue. Although, the problems faced by men due to anaemia are a little different in nature from women, they do hamper their life in general. Studies have shown that among adult males, iron deficiency anaemia hampers the productivity and physical capacity and the significant symptoms comprise of tiredness, lethargy and fatigue [10, 11]. A qualitative study conducted in the rural area of Haryana revealed that the knowledge and attitude toward dietary factors of anaemia were found to be conflicting among men. Despite being aware of the importance of good diet and green leafy vegetables, the study participants stated easy availability of dairy products and unaffordability of green leafy vegetables and fruits as the two main reasons of low intake of green leafy vegetables [12].

The National Policy of 1993 was a dedicated approach towards overall development and wellbeing of the citizens in India and took a bold step towards curbing the rates of anemia in the country. However, it focused upon improving the nutritional status of the most fragile and vulnerable section, i.e., women and children. Women and children were considered as nutritionally at risk and men were not even in the radar of this policy. Various national and state level policies have been formulated from time to time but they neglected male population in the country. For instance, Integrated Child Development Services (ICDS) in 1925 and Mid-Day Meal Scheme in 1962–63, the Special Nutrition Programme, Balwadi Nutrition Programme and National Anaemia Prophylaxis Programme in 1970–71, all were centered towards preventing anaemia among mothers and children of different age groups [13]. Similarly, the National Iron+ Initiative launched by the Adolescent Division of the Ministry of Health and Family Welfare (MoHFW), Government of India was intended to reduce anemia among the vulnerable groups such as pregnant women, infants, young children and adolescents by providing supplementation of Iron & Folic Acid [14]. Other initiatives which neglected men over women like Anaemia Mukt Bharat also were based on the idea of reducing of anemia prevalence among young children 6–59 months and women of the reproductive age group in India [15, 16].

Numerous studies have taken up the theme of addressing the gender gap in adult malnutrition in India [17, 18]. The surplus amount of studies have focused on the issues of anaemia among women, children and adolescents [17, 19, 20]. Either most of the studies on anaemia have always been limited to women and children, or they have been mostly conducted in the underdeveloped countries and developing countries, other than India [21–23]. Anaemia has been a constant issue of struggle among men, both in terms of prevalence and economic impact, it has received little research and policy attention [24]. There is a dearth of studies about the issue of anaemia among men in India.

Moreover, the existing few studies do not reveal much about the dynamics of associated factors regarding anaemia and the inherent socio-economic inequality in it. The policies and programmes catered to combat anaemia are focused on women and children and hence ignoring the men in policymaking. This study sought to examine the inequality in anaemia levels among men and to decompose the factors behind this process among the male population in India.

## Data and methods

### Data

National representative cross-sectional data from the fourth round of the National Family Health Survey (NFHS-4), conducted in India in 2015–16, was used for this study. The survey collected demographic, socioeconomic, and health information from a nationally representative probability sample of 699,868 women aged (15–49 years) and 112,122 men aged (15–54 years) with a response 97% and 92% respectively, residing in 601,509 households. NFHS-4 adopted a stratified two-stage sampling procedure and covered all 36 states and union territories. Full details of the survey have been published [5] and are available at https://dhsprogram.com/data/available-datasets.cfm. Additionally, the survey designed to provide vital estimates of clinical, anthropometric and biochemical (CAB) measurements; prevalence of malnutrition, anaemia, hypertension, HIV, and high blood glucose levels through a series of biomarker tests and measurements.

The Biomarker Questionnaire covered measurements of height, weight, and haemoglobin for children, and measurements of height, weight, haemoglobin, blood pressure, and random blood glucose for women aged 15–49 years and men aged 15–54 years (*in the state module subsample of households only*). The adequate sample size for this study was 108,261 men aged 15–54 years. We removed the missing information for better estimates.

### Outcome variable

#### Anaemia testing

In NFHS-4, blood specimens for anaemia testing were not collected by the authors but by trained health investigators with the consent of the respondents. Blood samples were drawn from a drop of blood taken from a finger prick and collected in a microcuvette. Haemoglobin analysis was conducted on-site with a battery-operated portable HemoCueHb 201+ analyser. More details on how haemoglobin was measured can be found in the national report http://rchiips.org/nfhs/pdf/NFHS4/India.pdf [5]. Following WHO’s recommendations, men were categorised as anaemic in any form if their haemoglobin concentration was lower than 13.0 g/dL, mildly anaemic if it was 12.0–12.9 g/dL, moderately anaemic if it was 9.0–11.9 g/dL, and severely anaemic if it was lower than 8.9 g/dL. For the analysis purpose, the study created a dichotomous variable coded as 1 ‘anaemia’ if men’s haemoglobin level was lower than 13.0 g.dL and 0 ‘not anaemic’ if the haemoglobin level was higher than 13.0 g.dL among men.

### Exposure variables

Given the dearth of research on men’s anaemia, the exposure variables were selected based on literature available on the current matter [12, 25]. These included age of men (15–19, 20–24, 25–29, 30–34, 35–39, 40–44, 45–49 and 50–54 years), marital status (never married and currently married), men’s educational level (no education, primary, secondary and higher), men’s exposure to mass media (how often they read newspapers, listened to the radio and watched television; responses on the frequencies were: almost every day, at least once a week, less than once a week,or not at all; men were considered to have any exposure to mass media if they had exposure to any of these sources and as having no exposure if they responded with ‘not at all’ for all the three sources of media), body mass index (underweight: < 18.5 kg/m^2^, normal: 18.5–24.99 kg/m^2^, and overweight: ≥25 kg/m^2^), tobacco use (if men used any forms of tobacco (smoke/smokeless) coded as ‘1’ yes and 0 ‘no’ otherwise), and caste [scheduled caste (SCs), scheduled tribe (STs), other backward class (OBC), and others (including all privileged caste groups)]. Other predictors were religion [Hindu, Muslim and others (including Christian, Sikh, Buddhist/Neo-Buddhist, Jain, Jewish, Parsi/Zoroastrian, no religion, and other)], and wealth index (poorest, poorer, middle, richer and richest). For the calculation of wealth index, households were given scores based on the number and kinds of consumer goods they own, ranging from a television to a bicycle or car, and housing characteristics such as source of drinking water, toilet facilities, and flooring materials. These scores were derived using principal component analysis. National wealth quintiles were compiled by assigning the household score to each usual (*de jure*) household member, ranking each person in the household population by their score, and then dividing the distribution into five equal categories, each with 20% of the population [5] . Place of residence was given as rural and urban in the survey. Geographical regions were categorized as North, Central, East, Northeast, West, and South.

### Statistical analysis

Descriptive statistics were used to show the distribution of the study population. Further, bivariate and multivariate logistic regression analysis was used to identify the factors associated with the outcome variable. A Chi-square test was performed to understand the association between outcome variable and predictors. When exploring the association between the prevalence of anemia and men’s background characteristics, for ordered categorical characteristics, such as wealth index and education level, Cochran Armitage trend test was used. The analysis of the dataset was carried out after assigning survey weight available in the data set. Moreover, Variance Inflation Factor (VIF) was estimated to check for multicollinearity and 10 is taken as the cut-off value [26].

### Concentration index

Existing literature on measuring socio-economic inequality on various dimensions uses Concentration Index and Concentration curve [27–29]; hence we tried to adopt those methodologies in the present paper. The wealth quintile was the critical variable to measure the economic status of the household. The study used a wealth score for decomposition analysis and the calculation of Concentration Index (CI). The study divided ranking into five equal categories, each comprising 20% population. Concentration Index (CI) and Concentration curve (CC) was calculated to measure the socio-economic inequality in anaemia among men in India. Concentration index represents the magnitude of inequality by measuring the area between the concentration curve and line of equality and calculated as twice the weighted covariance between the outcome and fractional rank in the wealth distribution divided by the variable mean.

The concentration index can be written as follows:

$$ \boldsymbol{C}=\frac{\mathbf{2}}{\boldsymbol{\mu}}\boldsymbol{\operatorname{cov}}\left({\boldsymbol{y}}_{\boldsymbol{i},}{\boldsymbol{R}}_{\boldsymbol{i}}\right) $$

Where C is the concentration index; *y*_*i*_ is the outcome variable index; ***R*** is the fractional rank of individual ***i*** in the distribution of socioeconomic position; ***μ*** is the mean of the outcome variable of the sample, and ***cov*** denotes the covariance [30]. The index value lies between − 1 to + 1.

If the curve lies above the line of equality, the concentration index takes a negative value, indicating a disproportionate concentration of inequality among the poor (pro-rich). Conversely, if the curve lies below the line of equality, the concentration index takes a positive value, indicating a disproportional concentration of inequality among the rich (pro-poor). In the absence of socioeconomic related inequality, the concentration index is zero. The value of CI quantifies the extent of socio-economic inequality. The larger the absolute value, the greater the inequalities.

### Decomposition of the concentration index

The study used Wagstaff decomposition analysis in decomposing the concentration index. Wagstaff’s decomposition demonstrated that the concentration index could be decomposed into the contributions of each factor to the income-related inequalities [31]. Each contribution is the outcome of the sensitivity of health concerning that socioeconomic factor and the extent of income-related inequality in that factor. Based on the linear regression relationship between the outcome variable *y*_*i*_, the intercept α, the relative contribution of *x*_*ki*_ and the residual error *ε*_*i*_

$$ {y}_i=\alpha +\sum {\beta}_k{x}_{ki}+{\varepsilon}_i $$

Where *ε*_*i*_ is an error term, given the relationship between *y*_*i*_ and *x*_*ki*_, the CI for y (C) can be rewritten as

$$ C=\sum \left(\frac{\beta_k{\overline{x}}_k}{\mu}\right){C}_k+\frac{GC\varepsilon}{\mu }/\mu $$

Where *μ* is the mean of *y*_*i*_, ${\overline{x}}_k$, is the mean of *x*_*k*_, *β*_*k*_ is the coefficient from a linear regression of outcome variable, *C*_*k*_ is the concentration index for *x*_*k*_ (defined analogously to C, and GC_ɛ_ is the generalized concentration index for the error term (*ε*_*i*_).

Here C is the outcome of two components: First, the determinants or ‘explained’ factors, which are equivalent to the weighted accumulation of the concentration indices of the regressor, where a one-unit change in the outcome variable is to be associated with the one-unit change in the explanatory variable. The explained factors indicate that the proportion of inequalities in the outcome (anaemia among men) variable is explained by the selected explanatory factors, i.e., x_k_. Second, a residual or ‘unexplained’ factor $\left(\frac{GC\varepsilon}{\mu }/\mu \right)$, indicating the inequality in health variable that cannot be explained by selected explanatory factors across various socioeconomic groups.

## Results

### Socioeconomic and demographic profile of the study population in India (Table 1)

About 17% of men belonged to 15–19 years’ age group, 64% were currently married, and 13% of men had no education. Majority of men (92%) had mass media exposure, one-fifth of men were underweight (BMI-less than 18.5 kg/m^2^), and about 46% of men used tobacco (smoke/smokeless). Nearly one-fifth of men belonged to scheduled caste, and 10% of men belonged to scheduled tribe caste. A higher proportion of men belonged to the Hindu religion, and around 63% of men lived in rural areas.

**Table 1:** Socio-economic and demographic profile of the study population by background characteristics, India, 2015–16

Variables	Percentage	Sample
**Men’s age (in years)**		
15–19	16.7	18,382
20–29	29.1	31,542
30–39	25.4	27,629
40–49	21.0	22,387
50–54	7.8	8321
**Marital status**		
Never married	36.0	39,692
Currently married	64.0	68,569
**Educational level**		
No Education	13.1	14,545
Primary	12.7	13,930
Secondary	57.2	63,027
Higher	17.1	16,759
**Mass media**		
No exposure	8.0	10,050
Exposure	92.0	98,211
**Body Mass Index**		
Underweight (< 18.5 kg/m2)	19.7	20,760
Normal (18.5–24.99 kg/m2)	60.7	68,238
Overweight (≥25 kg/m2)	19.5	19,093
**Tobacco use (smoke/smokeless)**		
No	54.2	55,024
Yes	45.8	53,237
**Caste**		
Scheduled caste	19.9	19,257
Scheduled tribe	8.9	19,354
Other backward class	43.8	42,101
Others	27.4	27,549
**Religion**		
Hindu	81.9	80,885
Muslim	12.8	14,733
Others	5.3	12,643
**Wealth index**		
Poorest	14.9	17,927
Poorer	19.0	22,579
Middle	21.2	23,562
Richer	22.1	22,467
Richest	22.8	21,726
**Place of residence**		
Urban	37.3	33,779
Rural	62.7	74,482
**Region**^**€**^		
North	14.1	23,777
Central	22.0	27,306
East	18.9	16,683
Northeast	3.3	13,924
West	18.1	11,725
South	23.7	14,846
**Total**	**100.0**	**108,261**

€North: Chandigarh, Haryana, Himachal Pradesh, Jammu & Kashmir, Delhi, Punjab, Rajasthan, Uttarakhand; Central: Chhattisgarh, Madhya Pradesh, Uttar Pradesh; East: Bihar, Jharkhand, Odisha, West Bengal; Northeast: Arunachal Pradesh, Assam, Manipur, Meghalaya, Mizoram, Nagaland, Sikkim, Tripura; West: Dadra & Nagar Haveli, Daman & Diu, Goa, Gujarat, Maharashtra; South: Andaman & Nicobar Islands, Andhra Pradesh, Karnataka, Kerala, Lakshadweep, Puducherry, Tamil Nadu, Telangana

### Prevalence of anaemia among men by background characteristics in India (Table 2)

The prevalence of anaemia was significantly higher among men who belonged to 15–19 (29%) and 50–54 years’ age group (30%). Moreover, it was lowest among those who belonged to the 20–29 years’ age group (18.9%). Men’s education and wealth quintile of the household had a negative association with the prevalence of anaemia. For instance, it was more prevalent among men who had no education (30.6%) and lowest among those who had higher education (16.5%). Similarly, the prevalence of anaemia was significantly higher among men who belonged to most impoverished families (32.5%), and it was lowest among those who belonged to wealthiest families (17.5%). Men who had no mass media exposure (32.2%) had a significantly higher prevalence of anaemia compared to those who had media exposure (22.5%). Anaemia was significantly higher among men who used tobacco (smoke/smokeless) (24.2%) than those who did not use (22.6%). Men who belonged to scheduled caste (24.3%) and scheduled tribe (32.4%) caste group had a higher prevalence of anaemia than other caste groups. The prevalence of anaemia was significantly higher among men who lived in rural areas (25.9%) compared to those who lived in urban counterparts (18.9%). Moreover, the prevalence of anaemia among men was highest in the East region, and it was significantly lower in the South region.

**Table 2:** Percentage distribution of anemia among men by background characteristics, India, 2015–16

Background Characteristics	Anaemia	***p***-value
**Men’s age (in years)**		*p* < 0.001
15–19	29.2	
20–29	18.9	
30–39	21.0	
40–49	24.9	
50–54	30.3	
**Marital status**		*p* < 0.001
Never married	23.6	
Currently married	23.2	
**Educational level**		*p* < 0.001
No Education	30.6	
Primary	25.8	
Secondary	23.1	
Higher	16.5	
**Mass media**		*p* < 0.001
No exposure	32.2	
Exposure	22.5	
**Body Mass Index**		*p* < 0.001
Underweight (< 18.5 kg/m2)	32.2	
Normal (18.5–24.99 kg/m2)	22.3	
Overweight (≥25 kg/m2)	17.4	
**Tobacco use (smoke/smokeless)**		*p* < 0.001
No	22.6	
Yes	24.2	
**Caste**		*p* < 0.001
Scheduled caste	24.3	
Scheduled tribe	32.4	
Other backward class	22.6	
Others	20.8	
**Religion**		*p* < 0.001
Hindu	23.6	
Muslim	21.3	
Others	23.6	
**Wealth index**		*p* < 0.001
Poorest	32.5	
Poorer	27.0	
Middle	23.0	
Richer	20.2	
Richest	17.5	
**Place of residence**		*p* < 0.001
Urban	18.9	
Rural	25.9	
**Region**^**€**^		*p* < 0.001
North	20.4	
Central	24.4	
East	31.5	
Northeast	24.9	
West	19.6	
South	20.2	
**Total**	**23.3**	

*p* < 0.001 based on chi-square test and Cochran Armitage trend test for ordered variables (education and wealth)

€North: Chandigarh, Haryana, Himachal Pradesh, Jammu & Kashmir, Delhi, Punjab, Rajasthan, Uttarakhand; Central: Chhattisgarh, Madhya Pradesh, Uttar Pradesh; East: Bihar, Jharkhand, Odisha, West Bengal; Northeast: Arunachal Pradesh, Assam, Manipur, Meghalaya, Mizoram, Nagaland, Sikkim, Tripura; West: Dadra & Nagar Haveli, Daman & Diu, Goa, Gujarat, Maharashtra; South: Andaman & Nicobar Islands, Andhra Pradesh, Karnataka, Kerala, Lakshadweep, Puducherry, Tamil Nadu, Telangana

### Estimates from logistic regression analysis for anaemia among men in India (Table 3)

With reference to men aged 15–19 years’ age group, the likelihood of anaemia was 30% and 23% less likely among men who belonged to 20–29 [OR: 0.70; CI: 0.66–0.73] and 30–39 years [OR: 0.77; CI: 0.74–0.84] age group respectively and 20% more likely among those who belonged to 50–54 years age group [OR: 1.20; CI: 1.11–1.29]. Moreover, men who had primary [OR: 0.90; CI: 0.86–0.95], secondary [OR: 0.83; CI: 0.79–0.87] and higher education [OR: 0.71; CI: 0.66–0.75] were 10%, 17% and 29% less likely to have anaemia respectively compared to men who had no education. The odds of anaemia were 7% significantly less likely among men who had mass media exposure [OR: 0.93; CI: 0.89–0.98] than those who had no exposure. The likelihood of anaemia was significantly higher among men who were underweight [OR: 1.51; CI: 1.46–1.57] and lowered among those who were overweight [OR: 0.80; CI: 0.77–0.84] compared to men who had normal body mass index. The odds of anaemia were 33% more likely among men who belonged to the scheduled tribe group [OR: 1.33; CI: 1.26–1.40] compared to those who belonged to other caste groups. Household wealth had a negative relationship with anaemia among men. For instance, the odds of anaemia decreased with the increase in wealth quintile. Men who belonged to rural areas [OR: 1.12; CI: 1.08–1.16] had 12% higher risk of anaemia than those who belonged to urban ones. Moreover, the likelihood of anaemia was 40% more likely among men who belonged to East region [OR: 1.40; CI: 1.33–1.47] and 25%, 13% and 7% less likely among those who belonged to Northeast [OR: 0.75; CI: 0.70–0.79], West [OR: 0.87; CI: 0.83–0.93], and South region [OR: 0.93; CI: 0.88–0.98] compared to those who belonged North region.

**Table 3:** Estimates from logistic regression analysis for anaemia among men by their background characteristics, India, 2015–16

Background Characteristics	OR [95% CI]
**Men’s age (in years)**	
15–19	**Ref.**
20–29	0.70***(0.66–0.73)
30–39	0.77***(0.74–0.84)
40–49	0.96(0.90–1.02)
50–54	1.20***(1.11–1.29)
**Marital status**	
Never married	**Ref.**
Currently married	0.99(0.944–1.032)
**Educational level**	
No Education	**Ref.**
Primary	0.90***(0.86–0.95)
Secondary	0.83***(0.79–0.87)
Higher	0.71***(0.66–0.75)
**Mass media**	
No exposure	**Ref.**
Exposure	0.93***(0.89–0.98)
**Body Mass Index**	
Underweight (< 18.5 kg/m2)	1.51***(1.46–1.57)
Normal (18.5–24.99 kg/m2)	**Ref.**
Overweight (≥25 kg/m2)	0.80***(0.77–0.84)
**Tobacco use (smoke/smokeless)**	
No	**Ref.**
Yes	0.92***(0.90–0.95)
**Caste**	
Scheduled caste	0.97(0.92–1.01)
Scheduled tribe	1.33***(1.26–1.40)
Other backward class	0.97*(0.93–1.00)
Others	**Ref.**
**Religion**	
Hindu	**Ref.**
Muslim	0.88***(0.84–0.92)
Others	0.97(0.92–1.02)
**Wealth index**	
Poorest	**Ref.**
Poorer	0.90***(0.86–0.94)
Middle	0.83***(0.79–0.87)
Richer	0.78***(0.73–0.82)
Richest	0.75***(0.70–0.80)
**Place of residence**	
Urban	**Ref.**
Rural	1.12***(1.08–1.16)
**Region**^**€**^	
North	**Ref.**
Central	1.01(0.97–1.06)
East	1.40***(1.33–1.47)
Northeast	0.75***(0.70–0.79)
West	0.87***(0.83–0.93)
South	0.93**(0.88–0.98)

OR: Odds Ratio; CI: confidence Interval; Ref: Reference category; ***p < 0.001; **p < 0.05; *p < 0.10

€North: Chandigarh, Haryana, Himachal Pradesh, Jammu & Kashmir, Delhi, Punjab, Rajasthan, Uttarakhand; Central: Chhattisgarh, Madhya Pradesh, Uttar Pradesh; East: Bihar, Jharkhand, Odisha, West Bengal; Northeast: Arunachal Pradesh, Assam, Manipur, Meghalaya, Mizoram, Nagaland, Sikkim, Tripura; West: Dadra & Nagar Haveli, Daman & Diu, Goa, Gujarat, Maharashtra; South: Andaman & Nicobar Islands, Andhra Pradesh, Karnataka, Kerala, Lakshadweep, Puducherry, Tamil Nadu, Telangana

### Estimates of decomposition analysis for the contribution of various explanatory variables for anaemia among men (Table 4)

Results from decomposition analysis revealed that wealth quintile explained 25.1% of the Socio-Economic Status (SES) related inequality followed by geographical regions of India (20.9%), body mass index (17.8%) and educational level (14.6%) for anaemia among men in India. Additionally, place of residence, caste and mass media exposure explained SES related inequality for anaemia among men in India.

**Table 4:** Estimates of decomposition analysis for the contribution of various explanatory variables for anaemia among men in India, 2015–16

Background Characteristics	Coefficient	Elasticity	CI	Absolute contribution to CI	Percentage contribution	%
**Men’s age (in years)**						
15–19**(Ref.)**						0.4
20–29	− 0.360***	− 0.020	0.010	0.000	0.7	
30–39	− 0.240***	−0.012	− 0.002	0.000	− 0.1	
40–49	− 0.042	− 0.003	0.016	0.000	0.2	
50–54	0.179***	0.003	0.043	0.000	−0.4	
**Marital status**						
Never married **(Ref.)**						0.0
Currently married	−0.013	0.001	−0.013	0.000	0.0	
**Educational level**						
No Education **(Ref.)**						14.6
Primary	−0.102***	− 0.003	− 0.278	0.001	−3.3	
Secondary	−0.184***	−0.019	0.030	−0.001	2.0	
Higher	−0.349***	−0.010	0.440	−0.005	15.8	
**Mass media**						
No exposure **(Ref.)**						2.7
Exposure	−0.068***	−0.014	0.054	−0.001	2.7	
**Body Mass Index**						
Underweight (< 18.5 kg/m2)	0.411***	0.016	−0.219	−0.003	11.9	17.8
Normal (18.5–24.99 kg/m2)**(Ref.)**						
Overweight (≥25 kg/m2)	−0.222***	−0.006	0.309	−0.002	5.9	
**Tobacco use (smoke/smokeless)**						
No **(Ref.)**						−2.2
Yes	−0.079***	−0.004	− 0.149	0.001	−2.2	
**Caste**						
Scheduled caste	−0.036	0.001	−0.157	0.000	0.3	8.6
Scheduled tribe	0.283***	0.006	−0.420	− 0.002	8.4	
Other backward class	−0.036*	0.001	0.014	0.000	−0.1	
Others **(Ref.)**						
**Religion**						
Hindu **(Ref.)**						−0.4
Muslim	−0.130***	− 0.003	0.038	0.000	0.3	
Others	−0.033	0.001	0.229	0.000	−0.7	
**Wealth index**						
Poorest **(Ref.)**						25.1
Poorer	−0.107***	−0.003	− 0.512	0.002	−5.9	
Middle	−0.187***	−0.006	− 0.110	0.001	−2.4	
Richer	−0.253***	−0.008	0.323	−0.003	8.8	
Richest	−0.287***	−0.009	0.772	−0.007	24.6	
**Place of residence**						
Urban **(Ref.)**						12.6
Rural	0.115***	0.016	−0.230	−0.004	12.6	
**Region**^**€**^						
North **(Ref.)**						20.9
Central	0.012	0.003	−0.161	−0.001	1.9	
East	0.334***	0.015	−0.339	−0.005	17.9	
Northeast	−0.294***	0.000	−0.253	0.000	0.4	
West	−0.134***	−0.002	0.156	0.000	1.1	
South	−0.070**	0.001	0.186	0.000	−0.3	
**Calculated CI**				**−0.029**		
**Actual CI**				**−0.121**		
**Residual**				**−0.092**		

CI: concentration Index; Ref: Reference category; ***p < 0.001; **p < 0.05; *p < 0.10; %: percentage

€North: Chandigarh, Haryana, Himachal Pradesh, Jammu & Kashmir, Delhi, Punjab, Rajasthan, Uttarakhand; Central: Chhattisgarh, Madhya Pradesh, Uttar Pradesh; East: Bihar, Jharkhand, Odisha, West Bengal; Northeast: Arunachal Pradesh, Assam, Manipur, Meghalaya, Mizoram, Nagaland, Sikkim, Tripura; West: Dadra & Nagar Haveli, Daman & Diu, Goa, Gujarat, Maharashtra; South: Andaman & Nicobar Islands, Andhra Pradesh, Karnataka, Kerala, Lakshadweep, Puducherry, Tamil Nadu, Telangana

**Fig. 2:**
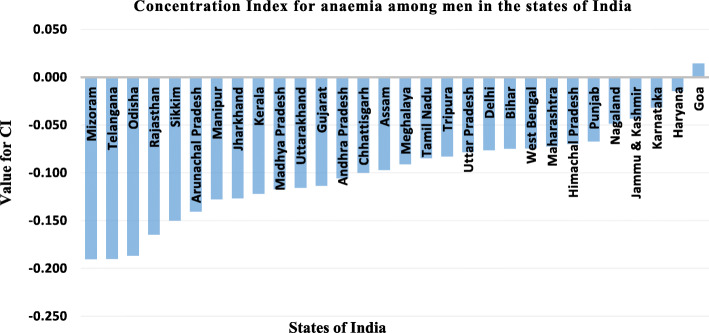
Concentration Index for anaemia among men in the states of India

Figure 2 depicts the value of concentration index for anaemia among Indian states. The figure craves out the fact that which state has the highest inequality in terms of anaemia among men, which as a whole is concentrated among men from poor wealth quintile. Highest inequality was witnessed in Mizoram (− 0.190) followed by Telangana (− 0.190), Odisha (− 0.187) and Rajasthan (− 0.165) whereas the lowest inequality was recorded in Goa (0.014), followed by Haryana (− 0.015), Karnataka (− 0.032) and Jammu & Kashmir (− 0.043).

**Fig. 3:**
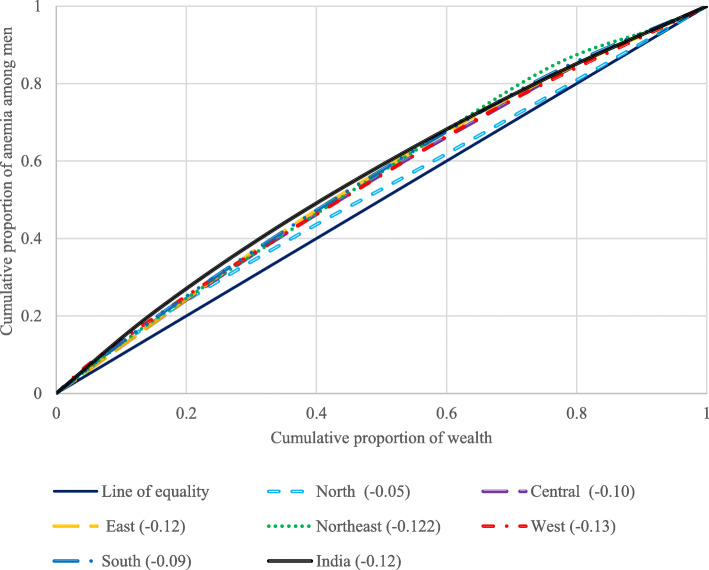
Concentration curve for anaemia among men in India by regions

Figure 3 depicts the concentration curve for anaemia among men across six geographical regions of India. The curve above the line of equality shows that anaemia was concentrated among men from low socioeconomic status. The negative value of the concentration index depicts that the outcome variable (anaemia among men) is concentrated among the poor. The value of CI for India was (− 0.12), which depicts pro-rich bias of anaemia among men. The highest inequality was witnessed in south and north-eastern region (− 0.11) followed by eastern and western region (− 0.10) of India, while lowest inequality was observed in north (− 0.05) and central (− 0.09) regions.

## Discussion

Anaemia is a major public health problem affecting 1·62 billion people worldwide. Although the prevalence of anaemia is estimated at 9% in countries with high development, in countries with low development the prevalence is 43% [4]. Research on anaemia in developing countries like Uganda [32], Bangladesh [33–35], Sri Lanka [36, 37] mostly concentrates on children and women. Studies in India are no different [19, 38] except a few [15, 25]. Hence, majorly overlooking men in the population and creating a void in the policies that focus on anaemia.

To overcome this, the present research investigates socioeconomic inequality in anaemia among men aged 15–54 years in India by utilizing the fourth round of the National Family Health Survey. The study found that men in the age group 50–54 years, with no education and mass media exposure, who were underweight (< 18.5 kg/m2), belonged to the schedule tribe, were from poorest wealth quintile, lived in rural areas, and eastern region of the country had a higher likelihood of anaemia. The results from decomposition analysis further strengthened these findings by showing the contribution of each of the socio-economic characteristics towards men having anaemia. For instance, the majority of the SES related inequality was explained by wealth quintile followed by geographical regions of India, body mass index and educational attainment.

A recent study on anaemia among men in India found similar findings on the socio-demographic factors at the individual level [25]. In rural Punjab, Gupta et al. found that both males and females who were underweight, and who belonged to a lower socio-economic status had higher prevalence of anaemia [39]. Research in China, Peru, Egypt, and the US suggest that a higher intake of vitamin C and iron by overweight and obese women might partly be the cause of the discrepancy between anaemia and BMI status [40–42]. However, since the results are based on women, any speculation about anaemia & BMI status of men should be interpreted with caution. A study in the US found that rates of anemia in men increased monotonically with age [43] . Further, a community-based cross-sectional survey in rural Haryana, India indicated a positive relationship between age of men and their anaemia prevalence [5]. An increase in proportion of normocytic anaemia might suggest the high prevalence of anaemia in male adults as their age increases [44].

The study findings specified the significant role of wealth quintile in SES related inequality in anaemia prevalence among men in India, and the finding is similar to another study [18]. In India, men belonging to the poorest wealth quintile from the states of Mizoram, Telangana, Odisha and Rajasthan suffer from the highest inequality in terms of anaemia. Further, the concentration index and concentration curve show that anaemia prevalence in men is highly concentrated among the poor in the north-eastern, eastern and western region of the country. The inter-relationships between poverty and malnutrition are well explained in previous studies. Evidence shows that malnutrition produces conditions of poverty by reducing the economic potential of the population and likewise, poverty reinforces malnutrition by increasing the risk of food insecurity [45]. Similarly in India, poverty restricts access to food that is required to meet daily requirements or to ensure dietary diversity, that leads to malnutrition, which again adversely affects educational and economic attainments, thus perpetuating poverty [46]. In fact, studies have shown the economic costs of anaemia in terms of lost schooling and lost productivity [7, 11]. Therefore, policymakers should include men belonging to poorest households and with low BMI in the target population to achieve an anaemia free India.

Education also plays a crucial role in explaining the SES related inequality. The present study revealed that men who had no education were more likely to be anaemic. The result is consistent with studies that examine similar results for women [32, 35, 39, 47]. Evidence indicates that education can change knowledge, attitudes, and practice, thereby improving nutritional status [48–52]. Moreover, a high level of individual educational also helps in being more receptive to the advice given by the health staff [53]. As individual level of education is important in addressing the problem of anaemia, so is awareness about it. For instance, results from a focus group discussion conducted on adult male members revealed that although men were aware of iron tablets used for treating anaemia but thought it was provided only to pregnant women by the health system [12]. To avoid such ignorance, WHO recommends health and other community infrastructures to organize comprehensive education and information program that motivates people to take iron tablets [54].

Despite the continuous effort of the Government of India to eliminate anaemia, the results emphasize that burden of anaemia exists in India. Previously, the government has undertaken various policies, for instance, the National Nutritional Anaemia Prevention Program, National Iron Plus Initiative, Anaemia Mukt Bharat. However, most of the policies focused on pregnant women, children and adolescents and hence the inequality. Our study results emphasize that older men also belong to the high-risk groups, and therefore the existing programs should be extended to include men. Community health workers (i.e., Accredited Social Health Activists) can promote awareness about anaemia through household visits and community meetings.

## Data Availability

The data used in the present study is available on https://dhsprogram.com/data/available-datasets.cfm.
